# Chemically synthesized CdSe quantum dots inhibit growth of human lung carcinoma cells via ROS generation

**DOI:** 10.17179/excli2015-705

**Published:** 2016-01-20

**Authors:** Aditya Kumar Jigyasu, Sahabjada Siddiqui, Mohatashim Lohani, Irfan Ali Khan, Md Arshad

**Affiliations:** 1 Department of Physics, Integral University, Lucknow-226026, India; 2 Molecular Endocrinology Laboratory, Department of Zoology, University of Lucknow, Lucknow-226007, India; 3 Department of Biosciences, Integral University, Lucknow-226026, India

**Keywords:** CdSe quantum dots, transmission electron microscope, A549, reactive oxygen species, DNA fragmentation

## Abstract

Quantum dots (QDs), semiconducting materials have potential applications in the field of electronic and biomedical applications including cancer therapy. In present study, cadmium selenide (CdSe) QDs were synthesized by chemical method. Octadecene was used as non-coordinating solvent which facilitated the formation of colloidal solutions of nanoparticles. CdSe QDs were characterized by UV-vis spectrometer and transmission electron microscope (TEM). The size measured by TEM was varied between 2-5 nm depending upon temperature. The cytotoxic activity of QDs was monitored by MTT assay, nuclear condensation, ROS activity and DNA fragmentation assay on human lung epithelial A549 cell line. Cells were treated with different concentrations of varying size of CdSe QDs for 24 h. CdSe QDs induced significant (*p *< 0.05) dose dependent cytotoxicity and this was comparable to the sizes of particles. Smaller particles were more cytotoxic to the large particles. Fluorescence microscopic analysis revealed that QDs induced oxidative stress generating significant ROS level and consequently, induced nuclear condensation and DNA fragmentation. Study suggested the cytotoxicity of CdSe QDs *via* ROS generation and DNA fragmentation depending upon particles size.

## Abbreviations

Abbreviations: CdSe, cadmium selenide; CNFs, carbon nanofiber; CNTs, carbon nanotubes, DAPI, 4', 6'-diamidino-2 phenylindole; DCFH-DA, 2,7-dichlorodihydrofluorescein diacetate; DMSO, dimethylsulfoxide; ELISA: enzyme linked immunosorbent assay; FBS, fetal bovine serum; MTT, 3-(4,5-dimethylthiazol-2-yl)-2,5-diphenyltetrazolium bromide; PI, propidium iodide; QDs, Quantum dots; ROS, reactive oxygen species; SEM, scanning electron microscope; SEM, standard error mean; TEM, transmission electron microscope

## Introduction

Cancer remains a global public health problem as the second leading cause of death in developed countries. Among cancer, lung carcinoma is the leading cause of cancer death in males due to its high incidence and mortality in both developed and developing countries (Torre et al., 2015[29]). Lung cancer is the most commonly diagnosed cancer with its annual death rate being over 1.3 million globally (Daga et al., 2015[7]). Therefore, the research is being investigated to eradicate and overcome these global health problems.

Rapid application of nanotechnology and nanomaterials represents a promising vista for the development of anti-cancer therapeutics. A large number of nanoparticles including organic and inorganic materials have been developed for delivery systems in cancer therapy since last two decades. Synthesis of various kinds of nanomaterials such as quantum dots (QDs), carbon nanofiber (CNFs), carbon nanotubes (CNTs), and fullerenes and their biological applications including cancer therapy have been developed more increasingly (Madani et al., 2013[16]; Ashfaq et al., 2014[3]; García-Hevia et al., 2014[9]; Shi et al., 2014[22]). QDs are attractive fluorophores for biomedical imaging and also biological tagging (Nune et al., 2009[18]). Quantum dots labelled with bevacizumab complexes has been used for *in vivo *imaging of tumors in xenograft model (Gazouli et al., 2014[12]). Cadmium selenide/cadmium telluride (CdSe/CdTe) QDs, when coated with lipids demonstrated extraordinarily high specificity for cancer cells (Shao et al., 2014[21]). CNFs and CNTs have gained tremendous attention as promising nanocarriers for delivery of small molecule including anticancer drugs (Ashfaq et al., 2014[3]; Wong et al., 2013[30]; Arlt et al., 2010[1]). Each of the components in CdSe QDs has *also* gained as a potent anticancerous agent. Since, a recent study showed that synthesized nano-Se attenuates cyclophosphamide-induced pulmonary injury through modulation of oxidative stress and DNA damage in Swiss albino mice (Bhattacharjee et al., 2015[5]). Selenium compounds act as therapeutic agents in cancer at higher doses that can turn into a prooxidant and thereby exert its potential anticancer properties (Fernandes and Gandin, 2015[8]). A study showed that Cd altered the cell cycle profile and apoptosis of human breast cancer MCF-7 cells, and its effects were found to be greater when used either alone or in combination with 5-FU compared with 5-FU alone (Asara et al., 2013[2]). Regardless of the various systemic therapies, there is a continuous need for research to develop novel anticancer therapeutics to improve clinical treatments and health problems.

Lung cancer cell line has been widely used as a tool for the development of biomedical drug discovery and research (Gazdar et al., 2010[11]). Therefore, in the present study, we selected A549 human lung carcinoma cells to study the cytotoxic nature of synthesized cadmium selenide (CdSe) nanoparticles. Small size of particles makes nanotechnology as a boon in medicine and industry. Interestingly, a research has revealed that smaller particles are more reactive and show their toxic behaviour due to intrinsic properties of nanoparticles and also the larger surface area to volume ratio (Gatoo et al., 2014[10]). Study revealed that nanoparticles react with the cells generating free radicals and cause oxidative stress leading to cell death (Smita et al., 2012[24]). Therefore, these allow us to synthesize and characterize the nanomaterials and subsequently i*n vitro* cell-based cytotoxicity studies in human lung carcinoma A549 cells.

In this study, CdSe QDs nanoparticles were prepared by chemical methods and the particles size and optical properties were investigated by UV-vis spectroscopy and transmission electron microscopy (TEM). In addition, we investigated *in vitro* cytotoxicity of CdSe QDs by MTT assay, reactive oxygen species (ROS) activity, nuclear condensation and DNA fragmentation assay on lung epithelial cell line A549. We observed that cadmium-based QDs were cytotoxic to lung cells which generate ROS and lead DNA damage. Our study suggested that the smaller QDs have stronger confinement making more cytotoxic than the large particles.

## Materials and Methods

### Chemicals and reagents

Cadmium oxide (CdO), oleic acid, selenium powder, trioctylphosphine, octadecene, Hoechst 33258, propidium iodide (PI), and 2,7-dichlorodihydrofluorescein diacetate (DCFH-DA) dye were purchased from Sigma Aldrich, USA. DMEM F-12 medium, Fetal bovine serum (FBS), MTT (3 (4,5-dimethylthiazol-2-yl) -2,5-diphenyltetrazolium bromide) dye, and antibiotic solution were purchased from Himedia, USA. All the reagents used in this study were of high purity grade.

### Chemical synthesis of quantum dots

CdSe QDs were synthesized using the standard protocol. In a typical procedure, 320 mg of Se powder was added to 3 ml octadecene solution in round bottom flask onto a stirrer hot plate. Approximately, 0.3 ml trioctylphosphine oxide was added in Se solution to dissolve the selenium completely. The prepared stock solution was used as a precursor for the preparation of different sizes of QDs. Approximately, 312 mg of cadmium oxide (CdO) was added with 0.6 ml oleic acid and 10 ml octadecene in a round bottom flask and cadmium solution was heated. When temperature reached at 240 °C, 1 ml of selenium solution was transferred to cadmium solution. The colour of this CdSe (QDs1) under the ultraviolet light was appeared blue in colour. The procedure was repeated at 250 °C temperature to produce different sizes of QDs (QDs2) which appeared green in colour. As the temperature was increased to 270 °C, the colour of QDs (QDs3) of different sizes was turned to red in colour.

### Isolation of CdSe QDs nanoparticles

This procedure was used to separate CdSe nanoparticles from the octadecene solvent. In brief, octadecene CdSe QDs suspension was resuspended with absolute ethanol. Samples were washed with ethanol by three times and centrifuged at 3,000 rpm for 5 min followed by removal of ethanol layer until no suspension was further obtained. Different sizes of QDs thus were isolated and resuspended in n-hexane solvent.

### Chemical characterization of CdSe QDs

CdSe QDs nanoparticles were characterized by UV-Vis spectroscopy and TEM. UV-Vis spectroscopy measurements were performed on a Shimadzu dual-beam spectrophotometer (model UV-1601 PC, Canada, USA) with a 1-cm quartz cell. Transmission electron microscopy was performed by drying a drop of suspension of CdSe nanoparticles onto a formvar coated TEM copper grids followed by analysis on Tecnai^TM^ G^2^ Spirit BioTWIN, (FEI, USA) equipped with Gaton orius Tm CCD camera controller which was operated at an accelerating voltage of 80 kV. For TEM characterization, samples were prepared by using 2 μl of QDs sample dissolved in 8 μl of n-hexane solvent and further bath sonicated for 10 min. Approximately, 4 μl of sample solution was putted on formvar coating grids and grids were then dried for 2-3 h in desiccators under vacuum. Thereafter, formvar coating grids were putted in a goniometer and electron micrographs were obtained.

### Cell line and culture

Human lung carcinoma A549 was purchased from cell repository-NCCS, Pune-India. A549 cell line was cultured in DMEM F-12 medium with 2.0 mM L-glutamine adjusted to contain 1.5 g/L NaHCO_3_ and 10 % fetal bovine serum. Cells were maintained at 37 °C, 5 % CO_2_ in a humidified air.

### In vitro MTT assay for cell viability

Cell viability assay of CdSe QDs nanomaterials were determined using MTT reduction assay following the protocol (Siddiqui and Arshad, 2014[23]). Cells were seeded at initial density 1x10^4^ cells/well of 96-well culture plate in 100 μl complete culture medium and incubated overnight. Stock solution of synthesized nanomaterials (QDs1, QDs2 and QDs3) was serially diluted into culture media to desired concentrations (0.5, 1, 10 and 25 µM) and added to the wells. After 21 h of treatment, 10 μl of a MTT solution was added and incubated for 3 h at 37 °C to generate purple formazan crystals. Formazan crystals were then solubilised in 100 μl of dimethyl sulfoxide (DMSO). Absorbance was recorded at 540 nm by a microplate ELISA reader (BIORAD Model 680, California, USA) and percent cell viability was calculated. 

### Reactive oxygen species (ROS) activity

Intracellular ROS generation was measured as per described previous protocol (Kaleem et al., 2015[14]). In brief, cells were exposed to small sizes of QDs (QDs1) at different concentrations (0.5, 1, 10 and 25 µM) for 12 h in 96-well culture plate. Cells were then incubated with DCFH-DA (10 mM) reagent and intracellular fluorescence intensity of cells was visualized by using inverted fluorescent microscope (Nikon ECLIPSE Ti-S, Tokyo, Japan).

For quantitative fluorometric analysis, cells were treated in 96-well black bottom culture plate for 12 h. After incubation with DCFH-DA (10 mM) solution at 37 °C for 30 min, fluorescence intensity was measured using a multiwell micro-plate reader (Synergy H1 Hybrid Multi-Mode Microplate Reader, BioTek, Vermont, USA) at an excitation wavelength 485 nm and emission wavelength 528 nm. Values were expressed as the percent of fluorescence intensity relative to the controls.

### Measurement of nuclear apoptosis by Hoechst-propidium iodide double stain

Hoechst-propidium iodide (PI) staining was used to analyse the apoptotic and necrotic cells after 24 h of above mentioned exposures as reported earlier (Tewari-Singh et al., 2012[28]). Briefly, at the end of treatment period, treated cells were washed twice with PBS and stained with 10 ml of PI (1 mg/ml) and Hoechst 33342 at a ratio of 3:1. For Hoechst 33342, the excitation wavelength is at 350 nm and emission at 461 nm and for PI excitation is at 535 nm and emission at 617 nm. Quantification of cells was performed in triplicate for each treatment and 100 cells per sample were counted in different fields to score the percent live, apoptotic and necrotic cells using a fluorescent microscope (Nikon ECLIPSE Ti-S, Tokyo, Japan). 

### DNA extraction and fragmentation

This assay was performed as described earlier with minor modifications (Ashfaq et al., 2013[4]). At the end of treatment period of 48 h, both treated and untreated cells were harvested and lyzed in 500 μl DNA lysis buffer (20 mM EDTA, 10 mM Tris-HCl pH 8.0, 0.2 % Triton X-100 and 100 μg/ml proteinase K) at 37 °C for 1.5 h. The lyzed cells were centrifuged at 6000 x g for 5 min. An equal volume of isopropanol and 25 ml 4M NaCl was added in the supernatant and incubated overnight at -20 °C. Samples were then centrifuged at 6000 x g for 30 min. The pellet was re-suspended in 50 μl ddH_2_O and 2 μl RNase A (10 mg/ml) at 37 °C for 1 h. Electrophoresis of extracted DNA was performed on 1.5 % agarose gel and DNA bands were observed under ultraviolet illumination gel-doc system (QUANTUMST4-1326.WL/26MX XPRESS, France).

### Statistical analysis

Data of cell viability were expressed as the mean ± SEM from three independent experiments. One-way ANOVA and Dunnett's Multiple Comparison Test was performed using Graph Pad prism (Version 5.0) software for significance test, using *p* value ≤ 0.05.

## Results and Discussion

### Optical study

**Figure 1:**
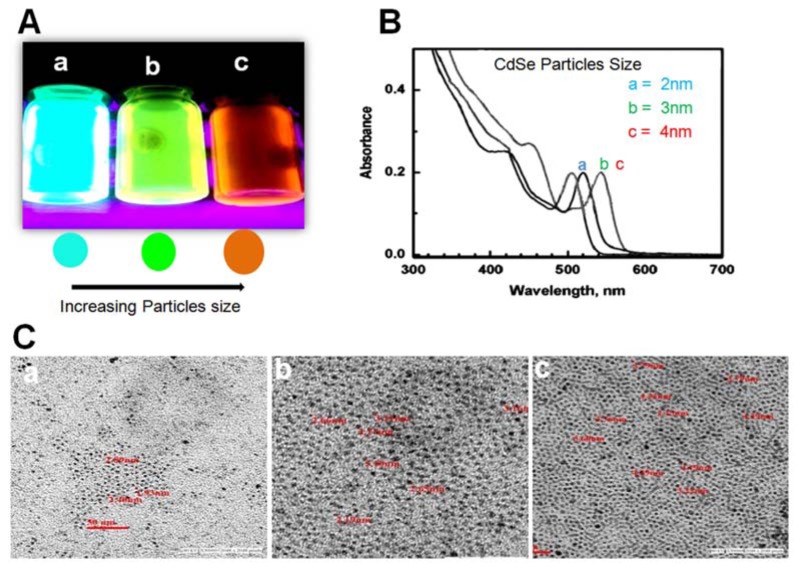
Chemical characterization of CaSe nanoparticles. (A) CdSe QDs of size 2 nm, 3 nm and 4 nm showing (a) blue (b) green and (c) red fluorescence under UV light (B) UV-vis absorption spectra of CdSe nanoparticles of (a) 2 nm (b) 3 nm and (c) 4 nm size (C) TEM images of CdSe QDs nanopaticles of (a) 2 nm (b) 3 nm and (c) 4 nm size.

CdSe quantum dots were successfully synthesized at various temperatures which appeared diverse in colour at different temperature under ultraviolet light. The prepared different sizes of QDs displayed very prominent fluorescence as shown in Figure 1A(Fig. 1). Results showed that QDs1 appeared blue in colour at 240 °C, QDs2 showed in green colour at 250 °C while QDs3 appeared red in colour at 270 °C under UV lamp. Previous studies have reported that change in colour is directly correlated with sizes of QDs (Gui et al., 2011[13]; Tamaki et al., 2014[26]). Interestingly, our synthesized CdSe QDs also affirm this argument. 

### Optical characterization of CdSe QDs by UV-vis spectrometer

The optical characterizations of QDs were performed at room temperature. The absorbance of different sizes of CdSe QDs was estimated by UV-vis spectrometry. As shown in the absorption spectra of Figure 1B(Fig. 1), a gradual shift was observed from shorter wavelength towards the longer wavelength with increase in particle size. A definite peak of smallest nanoparticle is confined at 500 nm. Another set of peaks were observed which shifted towards the longer wavelength as the particle size increased. This study showed that a blue shift in the first exciton peak position signal represented a decrease in CdSe core diameter while second and third exciton peak position showed an increase in CdSe core diameter. 

### Optical characterization of CdSe QDs by TEM

**Figure 2:**
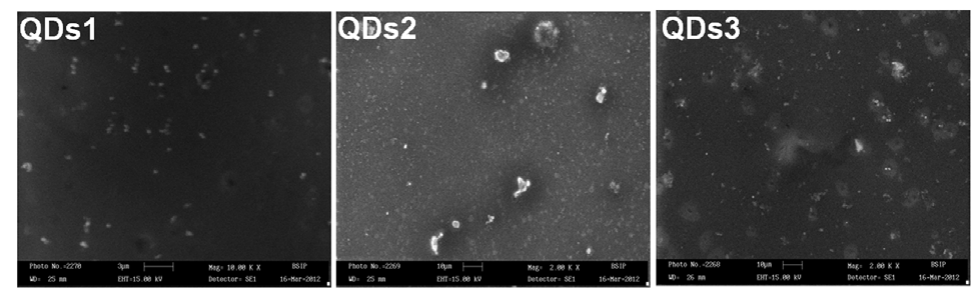
Chemical characterization of QDs by SEM. SEM images of CdSe QDs nanopaticles of (a) 2 nm (b) 3 nm and (c) 4 nm size

For surface studies of QDs, scanning electron microscope (SEM, LEO 430, England) analysis was performed. CdSe QDs were found spherical and round in shape (Figure 2(Fig. 2)). To further elucidate the particle size of QDs, TEM study was performed to record the TEM images of samples. The particle sizes were found in the range of 2-4 nm (Figure 1C(Fig. 1)). Interestingly, a similar study reported that the synthesized CdSe QDs, showed yellow to red fluorescence under UV light and the particle sizes were found in the range of 2.1-6.8 nm as confirmed by TEM (Surana et al., 2014[25]).

### In vitro antiproliferative activity

**Figure 3:**
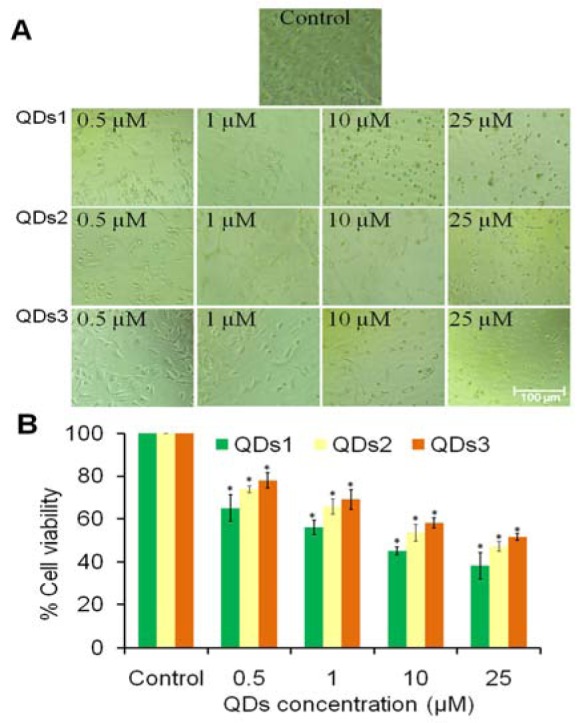
*In vitro *cytotoxicity of different sizes of CdSe QDs on A549 lung cancer cells by MTT assay. (A) Cellular morphology of A549 cells after 24 h treatment at different concentration (0.5-25 μM) of QDs by inverted phase contrast microscopy and (B) Dose-response effects of QDs on cytotoxicity against A54 cells. Scale bar=100 μm. Values are expressed as mean ± SEM of at least three independent experiments. *p < 0.05 as compared with their respective control.

The antiproliferative effect of nanomaterials was performed on A549 cell line using MTT cell viability assay. The effect of different concentrations of CdSe QDs1, QDs2 and QDs3 were evaluated and shown in Figure 3(Fig. 3). As shown in Figure 3A(Fig. 3), control (without treated) cells remained smooth, exhibiting normal cell surface, suggestive of their healthy nature. However, the cellular morphology of treated cells was changed displaying typical apoptotic features characterized by cellular shrinkage at different concentrations of QDs. The cytotoxic data indicated that 0.5 µM concentration of QDs1 reduced the cell viability to approximately 65 % (*p *< 0.05) as compared to control, which was drastically reduced to 56, 45.1 and 38.01 % (*p *< 0.05) at 1, 10 and 25 µM of QDs, respectively. Further, QDs2 at the concentrations of 0.5, 1, 10 and 25 µM reduced the cell viability to 73.93, 65.84, 53.63 and 47.17 % (*p *< 0.05), respectively. Similarly, QDs3 reduced the cell viability to 78, 69.06, 58.1 and 51.3 % (*p *< 0.05) at the concentrations of 0.5, 1, 10 and 25 µM, respectively. The cell growth inhibition data indicated that QDs significantly inhibited the growth of carcinoma cells in a dose dependent manner. The results from this study suggested that smaller size of QDs (QDs1) was more toxic to cells than larger size of QDs (QDs3). Interestingly, our data also support the results of the other study that smaller QDs displayed greater toxicity than larger QDs (Tang et al., 2013[27]).

### Reactive oxygen species (ROS) activity

**Figure 4:**
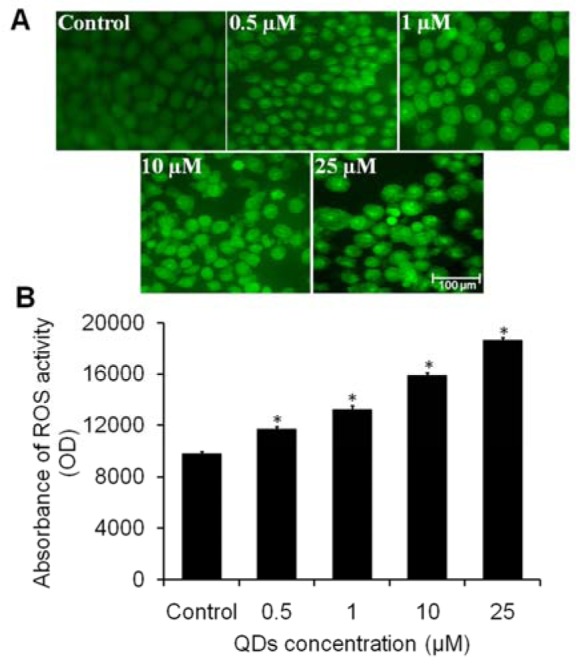
Effect of highly active CdSe QDs1 at different concentration (0.5-25 μM) on ROS production in A549 cells. (A) Photomicrographs showing intracellular ROS generation at different concentrations of QDs after 6 h incubation. Photomicrographs were taken with a florescence microscope. Scale bar=100 μm. (B) Graph showing extent of ROS generation with respect to the control. Values are expressed as the percentage of fluorescence intensity relative to the control. Values are expressed as mean ± SEM of at least three independent experiments, ^*^p < 0.05 as compared with their respective control.

Microscopic fluorescence imaging was used to study ROS generation in A549 cells after exposure to different concentrations of QDs. As observed from Figure 4A(Fig. 4), A549 lung cancer cells treated with QDs1 increased the ROS intensity in a dose dependent manner as compared to untreated cells. The results of quantitative measurement of ROS level also showed that higher dose of QDs induced the maximum ROS level compared to low doses of QDs. The qualitative data of ROS intensity was the consistent with the data of quantitative measurement of ROS level (Figure 4B(Fig. 4)). Study has suggested that oxidative stress plays an important role in the mechanism of toxicity for a number of nanoparticles through either the excessive generation of ROS or depletion of cellular antioxidant capacity (Manke et al., 2013[17]). ROS typically includes the superoxide radical, H_2_O_2_, and the hydroxyl radical, which damage cellular and nuclear components and lead to apoptotic cell death (Ott et al., 2007[19]). A further study has reported that GSH, a ubiquitous and abundant cellular antioxidant molecule is strongly depleted in response to nanoparticle exposure (Rana, 2008[20]). Based on these reports, our study could hypothesize that CdSe QDs might alter oxidant/antioxidant levels in A549 lung cancer cells by inducing ROS generation and declining the GSH level in A549 cells.

### Apoptosis by Hoechst 33258 and propidium iodide (PI) double staining 

**Figure 5:**
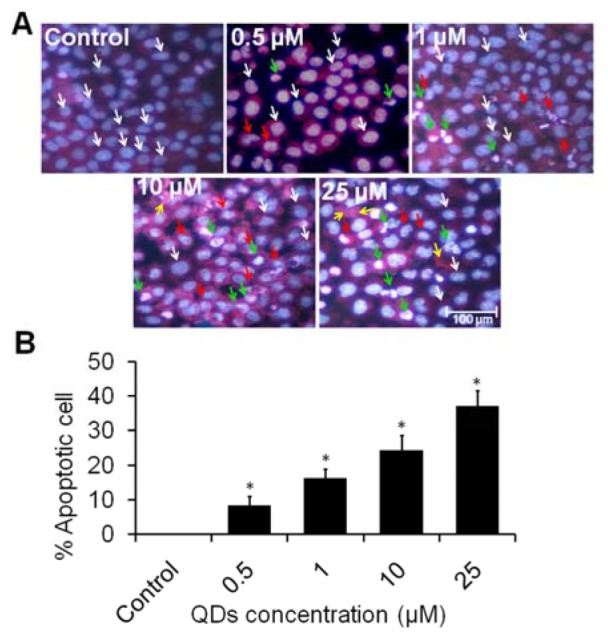
Effects of QDs1 on A549 cells inducing apoptotic cells and necrotic cells. (A) Photomicrographs showing apoptotic cells and necrotic cells treated with highly active QDs after 24 h incubation in A549 lung cell and evaluated by Hoechst 33258 and Propidium iodide (PI) double staining. Images were snapped with Nikon phase contrast microscope. White arrows, live cells - blue fluorescence and normal structure; red arrows, early apoptotic cells - bright blue florescence and enlarged with condensed or fragmented chromatin; green arrows, late apoptotic cells - bright red (pink to white) fluorescence in the centre due to condensed chromatin; yellow arrows, necrotic cells - bright red fluorescence and enlarged. (B) Representative graphs showing the quantified data in the form of percent apoptotic cells. Values are expressed as mean ± SEM of at least three independent experiments, ^*^p < 0.05 as compared with their respective control.

We further examined the overall effect on QDs-induced apoptotic and necrotic cell populations accompanied by a decrease in live cells. As observed from photomicrograph under inverted fluorescence microscope, chromatin condensation was increased dose dependently as compared to controls. Healthy cells showed blue fluorescence with normal structure while early apoptotic cells displayed bright blue florescence with fragmented chromatin. Late apoptotic cells showed pink to white fluorescence in the centre due to condensed chromatin and necrotic cells expressed bright red fluorescence (Figure 5A(Fig. 5)). This result supported the earlier study of the quantification of apoptotic and necrotic cell death in skin carcinoma cells (Tewari-Singh et al., 2012[28]). Furthermore, the quantification data demonstrated that approximately 8.33 % apoptotic cells were observed at 0.5 µM of QDs. The percent apoptotic cells were dramatically increased to 16.33, 24.33 and 37 % at 1, 10 and 25 µM of QDs, respectively (Figure 5B(Fig. 5)). Studies have demonstrated that presence of smaller DNA fragments reflect apoptotic cell death and higher fragmented DNA molecules indicates cell death due to necrosis (Rana, 2008[20]). Apoptosis results in fragmentation of cells into apoptotic bodies. Interestingly, the fragmented and condensed nuclei in A549 treated cells suggested that QDs induced cell death by an apoptotic process.

### DNA fragmentation analysis

**Figure 6:**
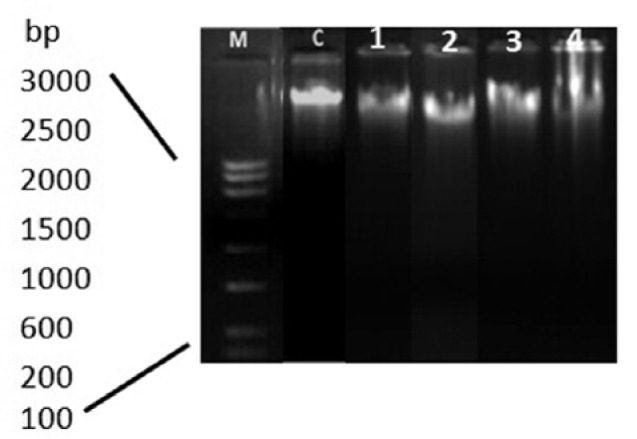
DNA fragmentation in A549 cells at different concentrations of QDs1 nanoparticles. Genomic DNA was isolated from A549 lung cancer cells. DNA ladders were visualized under UV light with ethidium bromide staining. Lane M: showing 3 kb DNA marker; Lane C: showing control A549 cells; Lane 1: showing cells treated with 0.5 μM; Lane 2: cells treated with 1.0 μM; Lane 3: cells treated with 1.0 μM; Lane 4: cells treated with 10 μM of QDs.

ROS act as an important regulator of apoptosis; however, overproduction of ROS in the cells results in the accretion of DNA damage indicating an early apoptosis (Linder et al., 2004[15]). Quantum dot was tested to ascertain DNA damage following its exposure to A549 cells for 24 h. Figure 6(Fig. 6) describes the results of DNA fragmentation analysis. The results for the control (untreated cells) showed undamaged DNA, represented by an intact band in the agarose gel. On the other hand, small shearing was observed in the cells treated with 0.5 μM, 1 μM and 10 μM QDs. However, maximum fragmentation was observed, when cells were treated with 25 μM of QDs. This fragmentation might be due to DNA damage caused by CdSe QDs presence in the nuclei. Study has been reported that free Cd^2+^ would lead to an increase in intracellular ROS levels which cause DNA damage and thus enhanced the cytotoxicity (Circu and Aw, 2010[6]).

In conclusion, the present study describes the synthesis and characterization of CdSe QDs nanoparticles and its *in vitro* cytotoxicity test mediated by induction of oxidative stress and subsequent activation of apoptotic cell death. Our results showed that the synthesized CdSe QDs induce a cyto-oxidative response in A549 cells depending upon particles size. This work will be very useful in destruction of cancerous cells and their therapy. Therefore, this type of QDs has the potential to be a promising alternative to cadmium-based anticancer agents.

## Notes

Sahabjada Siddiqui and Md Arshad (Molecular Endocrinology Laboratory, Department of Zoology, University of Lucknow, Lucknow- 226007, India; Tel.: +91-522-2370813; Fax: +91-522-2740230; e-mail: molendolab@gmail.com) contributed equally as corresponding authors.

## Acknowledgement

The authors extend their appreciation to Head, Department of Physics, Integral University for providing basic instrumentation facility and Head, Department of Zoology, University of Lucknow for cell culture facility. 

## Funding

Author AK Jigyasu is thankful to Department of Science and Technology [(No. DST/INSPIRE Fellowship/2011/(290)] for providing research grant. Author Sahabjada Siddiqui is thankful to Indian Council of Medical Research (ICMR), New Delhi, India for the award of SRF (No. 45/26/2013/ BMS/TRM). 

## Conflict of interest statement

None declared.
